# Gut microbiota composition during hospitalization is associated with 60-day mortality after severe COVID-19

**DOI:** 10.1186/s13054-023-04356-2

**Published:** 2023-02-23

**Authors:** Marius Trøseid, Jan Cato Holter, Kristian Holm, Beate Vestad, Taisiia Sazonova, Beathe K. Granerud, Anne Ma Dyrhol-Riise, Aleksander R. Holten, Kristian Tonby, Anders Benjamin Kildal, Lars Heggelund, Anders Tveita, Simen Bøe, Karl Erik Müller, Synne Jenum, Johannes R. Hov, Thor Ueland, Norwegian SARS-CoV-2 study group

**Affiliations:** 1 Research Institute of Internal Medicine, Oslo University Hospital, 0424 Oslo, Norway; 2 Section of Clinical Immunology and Infectious Diseases, Oslo University Hospital, 0424 Oslo, Norway; 3 Institute of Clinical Medicine, University of Oslo, 0315 Oslo, Norway; 4 Department of Microbiology, Oslo University Hospital, 0424 Oslo, Norway; 5 Department of Transplantation Medicine, Norwegian PSC Research Center, Oslo University Hospital, Oslo, Norway; 6 Department of Infectious Diseases, Oslo University Hospital, 0424 Oslo, Norway; 7 Department of Acute Medicine, Oslo University Hospital, Oslo, Norway; 8 Department of Anesthesiology and Intensive Care, University Hospital of North Norway, 9019 Tromsö, Norway; 9 Department of Clinical Medicine, Faculty of Health Sciences, UIT – The Arctic University of Norway, 9019 Tromsö, Norway; 10 Department of Internal Medicine, Drammen Hospital, Vestre Viken Hospital Trust, 3004 Drammen, Norway; 11 Department of Clinical Science, Faculty of Medicine, University of Bergen, 5009 Bergen, Norway; 12 Department of Internal Medicine, Bærum Hospital, Vestre Viken Hospital Trust, 1346 Gjettum, Norway; 13 Department of Anesthesiology and Intensive Care, Hammerfest County Hospital, Hammerfest, Norway; 14 Section of Gastroenterology, Department of Transplantation Medicine, Oslo University Hospital, Oslo, Norway; 15 K.G. Jebsen-Thrombosis Research and Expertise Center (TREC), UIT – The Arctic University of Norway, Tromsö, Norway

## Abstract

**Background:**

Gut microbiota alterations have been reported in hospitalized COVID-19 patients, with reduced alpha diversity and altered microbiota composition related to respiratory failure. However, data regarding gut microbiota and mortality are scarce.

**Methods:**

Rectal swabs for gut microbiota analyses were collected within 48 h after hospital admission (baseline; *n* = 123) and three-month post-admission (*n* = 50) in a subset of patients included in the Norwegian SARS-CoV2 cohort study. Samples were analysed by sequencing the 16S rRNA gene. Gut microbiota diversity and composition at baseline were assessed in relation to need for intensive care unit (ICU) admission during hospitalization. The primary objective was to investigate whether the ICU-related gut microbiota was associated with 60-day mortality.

**Results:**

Gut microbiota diversity (Shannon index) at baseline was lower in COVID-19 patients requiring ICU admission during hospitalization than in those managed in general wards. A dysbiosis index representing a balance of enriched and reduced taxa in ICU compared with ward patients, including decreased abundance of butyrate-producing microbes and enrichment of a partly oral bacterial flora, was associated with need of ICU admission independent of antibiotic use, dexamethasone use, chronic pulmonary disease, PO_2_/FiO_2_ ratio, C-reactive protein, neutrophil counts or creatinine levels (adjusted *p* < 0.001). The ICU-related dysbiosis index at baseline correlated with systemic inflammation and was associated with 60-day mortality in univariate analyses (Hazard ratio 3.70 [2.00–8.6], *p* < 0.001), as well as after separate adjustment for covariates. At the three-month follow-up, the dysbiosis index remained elevated in ICU patients compared with ward patients (adjusted *p* = 0.007).

**Conclusions:**

Although our data should be regarded as exploratory due to low number of clinical end points, they suggest that gut microbiota alterations during hospitalization could be related to poor prognosis after severe COVID-19. Larger studies of gut involvement during COVID-19 in relation to long-term clinical outcome are warranted.

*Trial registration* NCT04381819. Retrospectively registered May 11, 2020.

**Supplementary Information:**

The online version contains supplementary material available at 10.1186/s13054-023-04356-2.

## Introduction

Although SARS-CoV-2 primarily infects the respiratory tract, mounting evidence suggests that also the gastrointestinal (GI) tract is involved in the pathogenesis of COVID-19 [1]. SARS-CoV-2 infects human enterocytes and replicates in the gut mucosa [2], and viral entry receptors including angiotensin converting enzyme-2 (ACE-2) and several membrane-bound serine proteases are expressed in intestinal epithelial cells [3]. In a longitudinal study, viral shedding from faeces was detected in nearly 50% of patients during acute infection and in 3.8% of patients after 7-month follow-up [4].

Gastrointestinal symptoms are frequently occurring in severe COVID-19 patients, and a meta-analysis has suggested that patients with gastrointestinal involvement had a higher risk of severe disease [5]. It has also been hypothesized that the gut microbiota could be a mediator of host inflammatory immune responses during COVID-19, thereby contributing to the pronounced systemic inflammation observed in patients requiring hospitalization [6, 7].

Patients hospitalized with COVID-19 exhibit an altered gut microbiota composition compared to uninfected controls [8] and also compared to patients admitted with seasonal influenza [9]. Gut microbiota alterations, including reduced bacterial alpha diversity and changes in microbial composition, have been found to be related to disease severity, including acute respiratory failure and need of ICU admission (reviewed in [10]). We recently reported that the gut microbiota composition remained altered three months after hospitalization and was associated with persistent pulmonary pathology [11].

It has been hypothesized that COVID-related gut microbiota alterations (dysbiosis) could promote long-term clinical outcome [12]. A recent study of critically ill COVID-19 patients based on selective bacterial cultures and subsequent sequencing reported that higher concentrations of opportunistic pathogens in the oropharyngeal and intestinal compartments were independently associated with 90-day mortality [13]. However, data on sequencing-based gut microbiota dysbiosis and mortality are lacking. In the present substudy from the longitudinal Norwegian SARS-CoV2 cohort, we assessed gut microbiota alterations from samples collected within 48 h after hospitalization in relation to need for ICU admission and investigated whether an ICU-related dysbiosis was associated with 60-day mortality.

## Methods

### Patients and clinical outcomes

Study participants were recruited from the Norwegian SARS-CoV-2 study (NCT04381819), a multicentre cohort study of COVID-19 patients admitted to five Norwegian hospitals, conducted as part of an International Severe Acute Respiratory and Emerging Infection Consortium (ISARIC) WHO Clinical Characterization Protocol study, as previously described [14]. Participants aged ≥ 18 years and admitted to hospital with PCR-confirmed SARS-2-CoV-2 infection were eligible for inclusion and were included from March 9, 2020, until December 15, 2020. The study was approved by the Committee for Medical Research Ethics Region South-East Norway (approval no. 106624). All participants gave informed consent prior to inclusion, either directly or through a legally authorized representative. Gut microbiota alterations were assessed in relation to need for treatment at ICU/high-dependency unit during hospitalization, and the primary end point was 60-day post-admission all-cause mortality [14].

### Gut microbiota analyses

Rectal swabs were sampled within 48 h after admission and three-month post-admission, stored in a stabilizing transportation medium (soluble Amies, Thermo Scientific™) and frozen at − 80 °C until analysis. Faecal DNA was extracted using the QIAamp PowerFecal®Pro DNA Kit (Qiagen, Germany), with slight modifications. Briefly, 700 µL of faecal material was pelleted and homogenized in 800 µL of kit solution CD1 using a bead-beating homogenizer (2 × 60 s at 5.5 ms, 20 °C) and further processed according to the manufacturer’s protocol. Libraries for 16S rRNA amplicon sequencing were generated as previously described [11]. Briefly, the hypervariable regions V3 and V4 of the 16S rRNA gene were amplified using dual-indexed universal primers 319F (forward) and 806R (reverse), and Phusion High-Fidelity PCR Master Mix with HF buffer (Thermo Fisher Scientific, USA). The PCR products were cleaned and normalized using a SequalPrep Normalization Plate Kit (Thermo Fisher Scientific, USA). Quality control and quantification of the pooled libraries were performed using an Agilent Bioanalyzer (Agilent Technologies, USA) and Kapa Library Quantification Kit (Kapa Biosystems, London, UK). Sequencing was performed at the Norwegian Sequencing Centre (Oslo, Norway) using the Illumina MiSeq platform and v3 kit (Illumina, San Diego, CA, USA), allowing for 300 base pair paired-end reads.

### Sequence processing and bioinformatics

Paired-end reads containing Illumina Universal Adapters or PhiX were discarded using bbduk version 38.90 (https://sourceforge.net/projects/bbmap/) and the remaining reads were demultiplexed using cutadapt version 3.5 [15]. Trimming of indexes, heterogeneity spacers and primers was also done with cutadapt (parameters: -e 0.1 –overlap 20 –discard-untrimmed -m 250) and the paired-end reads were subsequently quality trimmed and merged using bbmerge version 38.90 [16]. The merged contigs were trimmed to 400 bp and denoised to ASVs (Amplicon Sequence Variants) with deblur in Qiime2 version 2022.2 [17]. Taxonomic classification of ASVs was done in Qiime2 using a naïve Bayes classifier [18] trained on the V3-V4 region of a preclustered version (99% sequence similarity) of the Silva database version 138 [19]. ASVs from mitochondria, chloroplast or lacking taxonomic annotation on order level were removed. Filtering of contaminants was done with the R package microDecon based on four negative control samples. A de-novo phylogenetic tree was built in Qiime2 based on the remaining ASVs. Differential abundance analysis was performed on genera present in at least 10% of patients. Gut microbiota alpha diversity was assessed by Shannon diversity index, performed on a rarefied (subsampled) dataset with an ASV count of 5144 per sample.

### Statistical analyses

Patient characteristics were compared using Student's t test or Mann–Whitney U-test depending on the distribution or chi-square for continuous and categorical variables, respectively. We first characterized gut microbiota alterations in relation to need for ICU treatment during hospitalization. Based on the differences (Table 1) between ward and ICU patients, and due to limited number of patients in the ICU group, we constructed a propensity score using logistic regression for adjustment purposes. Age was also included in the score due to near significant differences between groups. As several biochemical markers may reflect similar processes, C-reactive protein (CRP), neutrophil counts and creatinine were used in the propensity score. Similarly, we chose the PO_2_/FiO_2_ ratio over variables reflecting use of oxygen therapy. PO_2_/FiO_2_ is defined as the ratio between the partial pressure of oxygen in blood (PaO_2_) and fraction of inspired oxygen the patient is receiving (FiO_2_). All together, the propensity score consisted of age, use of dexamethasone or antibiotics, chronic pulmonary disease, PO_2_/FiO_2_ ratio, neutrophil counts, CRP and creatinine.

**Table 1:** Baseline characteristics in hospitalized COVID-19 patients according to outcomes

Parameter	Ward *n* = 95	ICU *n* = 28	*p*	Survivors *n* = 112	60-day mortality *n* = 11	*p*
Age, years	57 ± 15	62 ± 13	0.064	57 ± 14	67 ± 10	0.030
Male gender (%)	56 (59%)	19 (68%)	0.40	67 (59%)	9 (82%)	0.14
BMI, kg/m^2^	28.6 ± 4.6	29.6 ± 4.5	0.34	28.8 ± 4.7	28.9 ± 4.7	0.94
Dexamethasone	16 (17%)	18 (64%)	< 0.001	24 (21%)	10 (91%)	< 0.001
Antibiotics	25 (26%)	24 (86%)	< 0.001	39 (35%)	10 (91%)	< 0.001
*Comorbidities*						
Chronic cardiac disease (%)	20 (21%)	9 (32%)	0.22	24 (21%)	5 (46%)	0.073
Hypertension (%)	41 (43%)	14 (50%)	0.51	50 (46%)	4 (36%)	0.56
Chronic pulmonary disease (%)	16 (17%)	9 (32%)	0.077	21 (19%)	4 (36%)	0.16
Obesity (%)	25 (26%)	8 (29%)	0.81	29 (26%)	4 (36%)	0.49
Diabetes (%)	27 (29%)	6 (21%)	0.43	31 (28%)	2 (18%)	0.48
Cancer (%)	2 (3%)	1 (4%)	0.66	3 (3%)	0 (0%)	0.58
Current smoker (%)	5 (5%)	2 (7%)	0.71	7 (6%)	0 (0%)	0.39
*Oxygen therapy*						
Any oxygen therapy	52 (55%)	25 (89%)	0.001	68 (61%)	9 (82%)	0.17
Non-invasive ventilation	0 (0%)	7 (25%)	< 0.001	2 (2%)	5 (46%)	< 0.001
Invasive mechanical ventilation	0 (0%)	4 (14%)	< 0.001	3 (3%)	1 (9%)	0.25
*P/F-ratio at admission, kPa*	41.3 (34.3, 46.9)	21.7 (13.8, 38.2)	< 0.001	40.0 (31.0, 45.7)	14.0 (9.1, 42.7)	0.009
*Laboratory analysis at admission:*						
Haemoglobin, g/dL	13.3 ± 1.4	12.5 ± 1.9	0.016	13.2 ± 1.5	12.2 ± 1.6	0.052
C-reactive protein, mg/L	41 (15, 78)	82 (50, 119)	0.001	47 (18, 87)	95 (50, 142)	0.021
Ferritin, µg/L	498 (247, 837)	1090 (543, 1593)	< 0.001	541 (258, 882)	1096 (455, 2638)	0.018
White blood cell count, × 10^9^/L	6.2 ± 2.8	7.9 ± 3.6	0.011	6.6 ± 3	6.5 ± 3.5	0.94
Neutrophil count, × 10^9^/L	4.4 ± 2.6	6.8 ± 3.2	< 0.001	4.9 ± 2.9	6 ± 3.3	0.24
Lymphocyte count, × 10^9^/L	1.3 ± 0.6	0.9 ± 0.4	0.001	1.2 ± 0.6	0.7 ± 0.3	< 0.001
Creatinine, µM	75 ± 24	90 ± 42	0.022	77 ± 27	100 ± 42	0.014

Continuous data are given as mean ± SD or median (25th, 75th) percentile. *ICU* Intensive care unit, high-dependency unit was also classified as ICU

Differential abundance analysis was performed in baseline samples with ANCOM-BC2 [20] between ICU and ward patients. An ICU-related dysbiosis index representing a balance of enriched and reduced taxa in baseline faecal samples from ICU compared with ward patients was calculated by using the selbal algorithm (in R), which takes the compositional nature of microbiota data into account. Both the Shannon diversity index and ICU-related dysbiosis index were compared by linear regression adjusting for the propensity score. The distribution of Shannon diversity and ICU-related dysbiosis index are shown in Additional file 1: Fig. S1. The dysbiosis index showed a normal distribution but Shannon diversity was somewhat right skewed. However, log or log(*x* + 1) transformation rendered the variable more skewed and it was therefore used non-transformed.

We next investigated the association between the ICU-related dysbiosis index and 60-day all-cause mortality by receiver operating characteristic (ROC) analysis and Kaplan–Meier analysis (tertiles). The modulation of the association between dysbiosis index (as continuous variable) and mortality was further assessed in cox regression adjusting separately for covariates with trend level differences between survivors and non-survivors (Table 1) as well as for the ICU propensity score.

Statistical analysis was performed with IBM SPSS statistics package (version 27.0.0.0) and R: A language and environment for statistical computing (version 4.2.2).

## Results

### Baseline characteristics

**Fig. 1:**
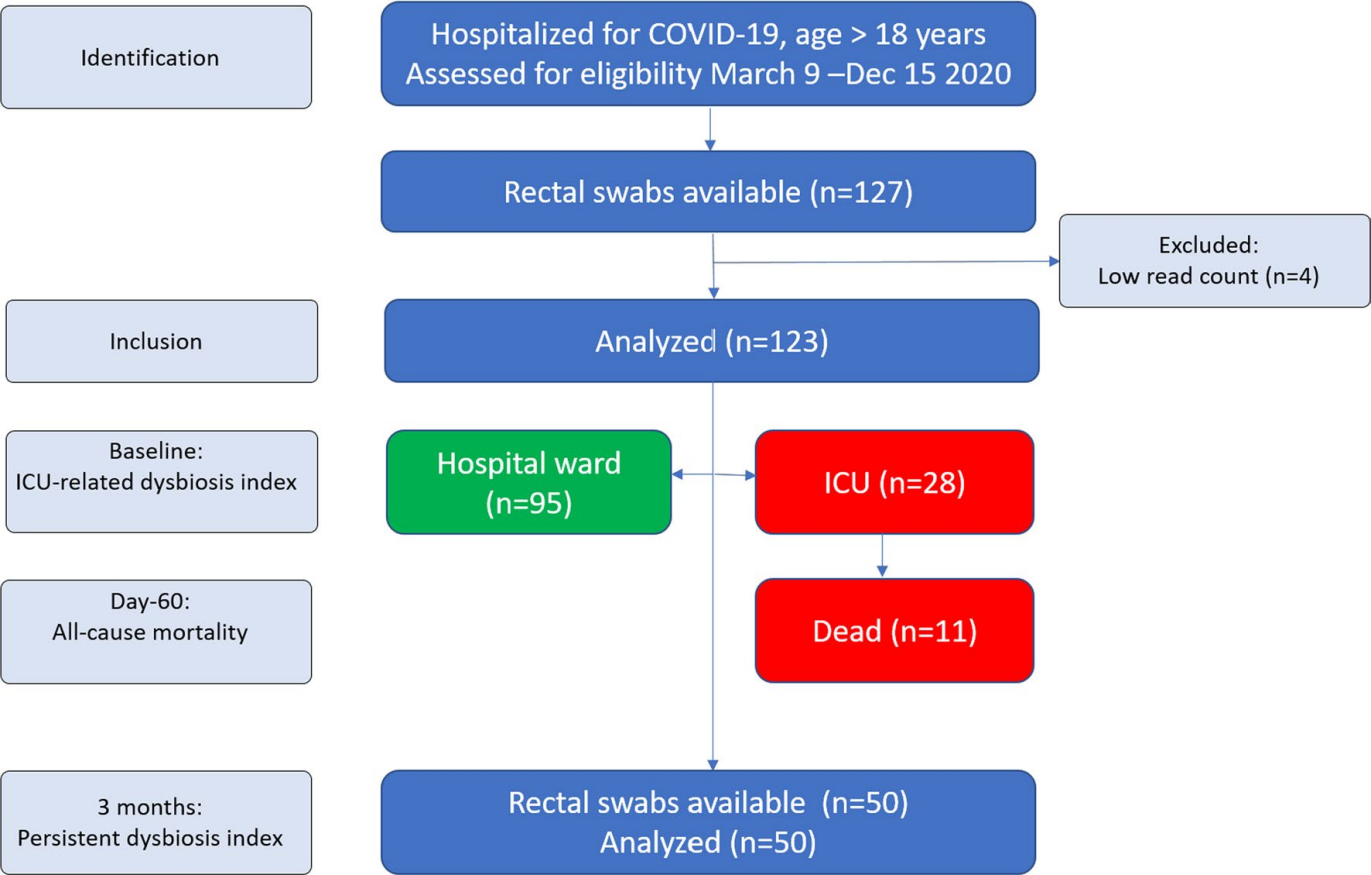
Flow chart of included participants (*n* = 123)

As shown in Table 1, patients were predominantly male, and 28 out of 123 participants were treated in the ICU during hospitalization. ICU patients were on average five years older than ward patients, with higher prevalence of pre-existing pulmonary disease, higher levels of systemic inflammatory markers and more often receiving antibiotics and dexamethasone treatment. Eleven participants (8.9%) died within 60 days of inclusion, and all of these were ICU patients, as shown in the flow chart (Fig. 1).

### Reduced alpha diversity and altered gut microbiota composition in ICU patients

**Fig. 2:**
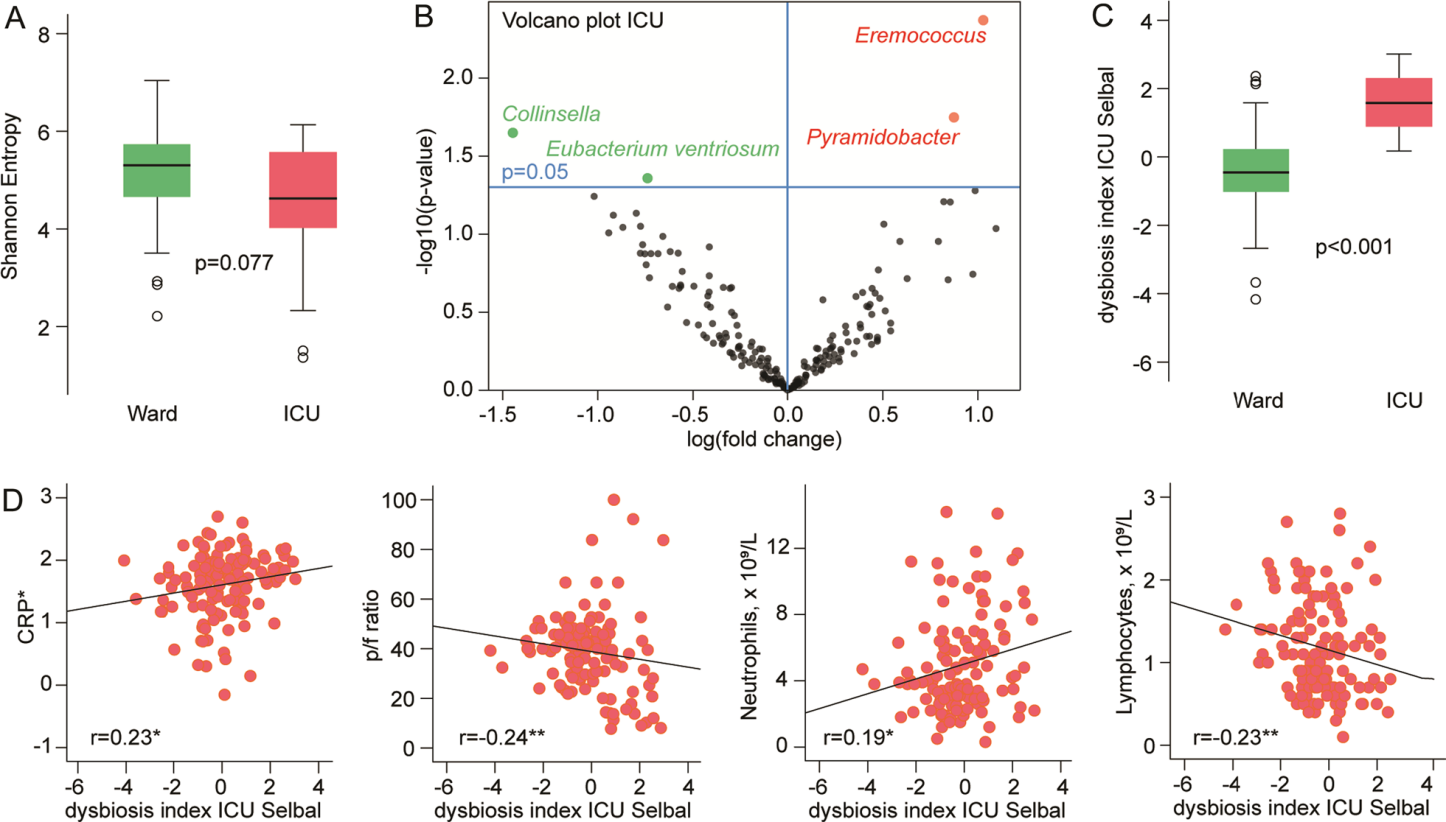
Gut microbiota alterations within 48 h after hospital admission in COVID-19 patients admitted to ICU (*n* = 28) compared to ward patients (*n* = 95). **A** Tukey plots showing reduced Shannon diversity index in ICU patients with adjusted *p* value (ICU propensity score, see statistical methods), **B** Volcano plot visualizing differentially abundant bacterial genera (from ANCOM-BC2) based on effect size (*x*-axis) and *p* values (*y*-axis) in relation to ICU status, **C** Tukey plot showing dysbiosis index for ICU vs. ward patients with adjusted *p* value (ICU propensity score, see statistical methods), **D** Correlations between dysbiosis index, degree of respiratory distress (*p*/PF-ratio) and inflammatory markers

As depicted in Fig. 2A, gut microbiota diversity assessed by Shannon diversity index at baseline was trend level lower (*p* = 0.077) in COVID-19 patients requiring ICU during hospitalization compared to ward patients in analysis adjusting for the ICU propensity score (see methods, statistical analysis, for definition). Shannon diversity was not significantly different in patients treated or not treated with dexamethasone or antibiotics (Additional file 1: Table S1). ICU patients also had an altered gut microbiota composition, with increased relative abundance of *Pyramidobacter* and *Eremococcus*, and reduced abundance of *Collinsella* and *Eubacterium ventriosum* group (Volcano plot, Fig. 2B).

### Dysbiosis index characterizing ICU patients independent of relevant confounders

For further analyses, we generated an ICU-related dysbiosis index based on the R package Selbal for selecting bacterial taxa distinguishing ICU from ward patients [21]. The following taxa were identified as enriched in ICU patients: *Pyramidobacter, Eremococcus, Ruminococcaceae;g_uncultured, Rothia, Intestinibacter, Olsenella, Fournierella,* and *Eubacterium eligens* group. The following taxa were reduced: Oscillospiraceae family, *Eubacterium ventriosum* group, *Fastidiosipila, Christensenellaceae;g__uncultured, Roseburia,* Alloprevotella, Terrisporobacter, *Incertae_Sedis;g__uncultured, Eubacterium siraeum* group, and *Howardella*. The index is defined as the difference between the arithmetic means of the log-transformed abundances in these two groups of taxa [21]. Only genera present in at least 20% of samples were considered for inclusion in the index. As shown in Fig. 2C, this index was associated with need of ICU (*p* < 0.001) independent of the ICU propensity score.

### ICU-related dysbiosis index in relation to degree of respiratory distress and inflammatory markers

As shown in Fig. 2D, the ICU-related dysbiosis index correlated negatively with the PO_2_/FiO_2_ ratio, with lower values reflecting increasing degree of respiratory failure. The dysbiosis index correlated positively with levels of C-reactive protein (CRP) and neutrophil count, and negatively with lymphocyte count. Overall, patients treated with dexamethasone and antibiotics had a higher ICU-related dysbiosis index (Additional file 1: Table S1).

### ICU-related dysbiosis index associated with 60-day mortality

**Fig. 3:**
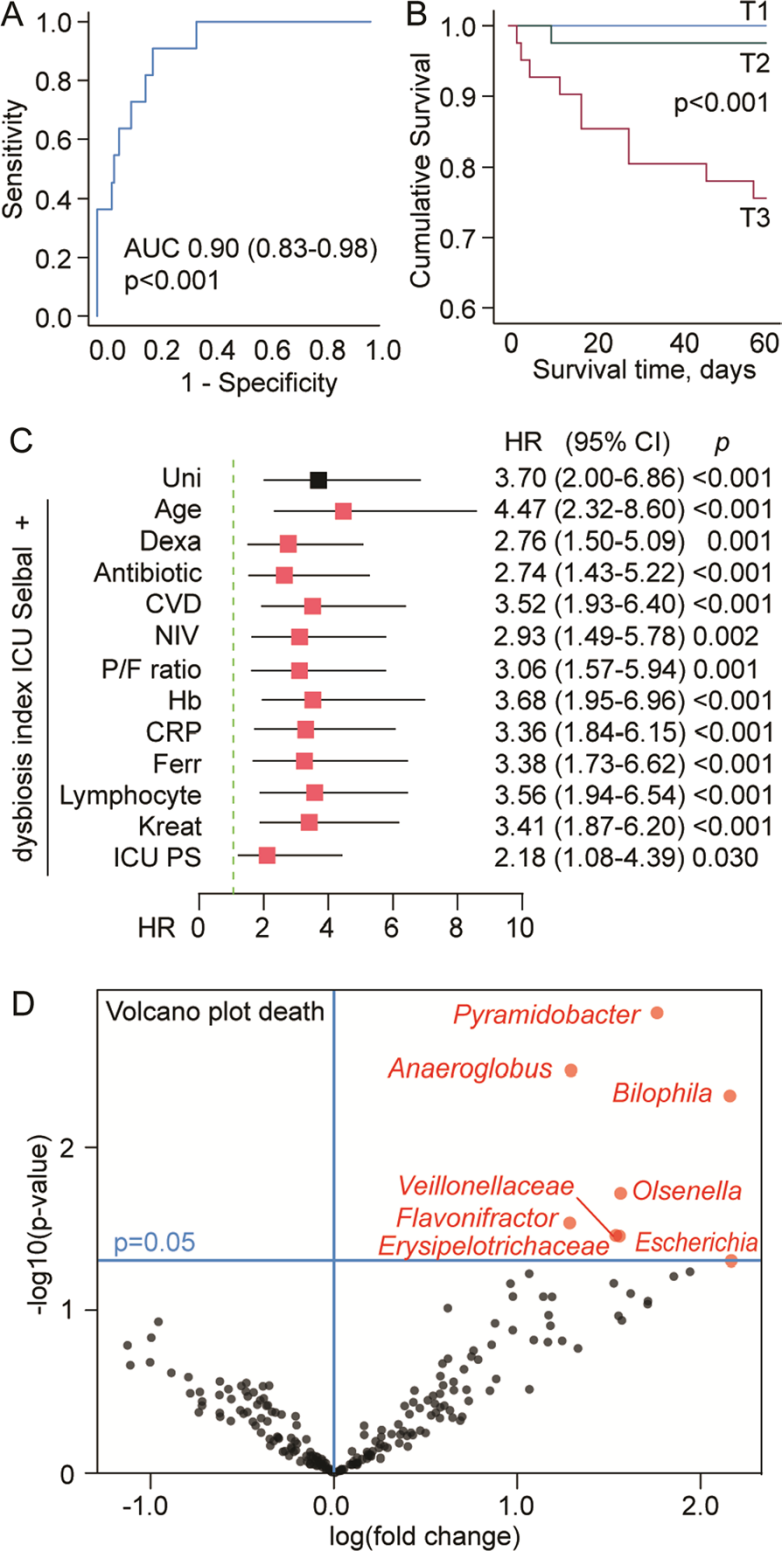
Association between gut microbiota alterations (dysbiosis) within 48 h after hospital admission and 60-day mortality in hospitalized COVID-19 patients. **A** ROC analysis of ICU dysbiosis index for 60-day mortality (*n* = 11). **B** Kaplan–Meier curve for 60-day mortality according to tertiles of ICU dysbiosis index. **C** Cox regression analysis of ICU dysbiosis index (as a continuous variable) in univariate analysis (black) and adjustment with covariates (red) one by one that were trend level different between survivors and non-survivors (see Additional file 1: Table S1, as well as ICU propensity score, see statistical methods), **D** Volcano plot visualizing differentially abundant bacterial genera based on effect size (*x*-axis) and *p* values (*y*-axis) in relation to 60-day mortality

During 60-day follow-up, 11 patients died. As shown in Kaplan–Meier plots in Fig. 3, the upper tertile of the dysbiosis index was associated with higher 60-day mortality (*p* < 0.001). A ROC analysis of the ICU-related dysbiosis index for 60-day mortality revealed an area under curve (AUC) of 0.90 (0.83–0.98), *p* < 0.001. Cox regression analysis of the dysbiosis index (as a continuous variable) was associated with 60-day mortality in univariate analyses (Hazard ratio 3.70 [2.00–8.6], *p* < 0.001). Clinical characteristics for survivors and non-survivors are given in Table 1, and adjusting separately for markers that were trend levels different between survivors and non-survivors did not alter this association (Fig. 3C). Adjustment with the ICU propensity score attenuated the association somewhat, but remained significant. Some of the microbes enriched in the ICU-related dysbiosis index, *Pyramidobacter* and *Olsenella*, in addition to bacteria previously reported in severe COVID-19, including Veillonellaceae, *Bilophila* and *Escherichia* [7] were also enriched in patients with a mortality end point (Fig. 3D).

### Persistent dysbiosis in ICU patients three-month post-admission

**Fig. 4:**
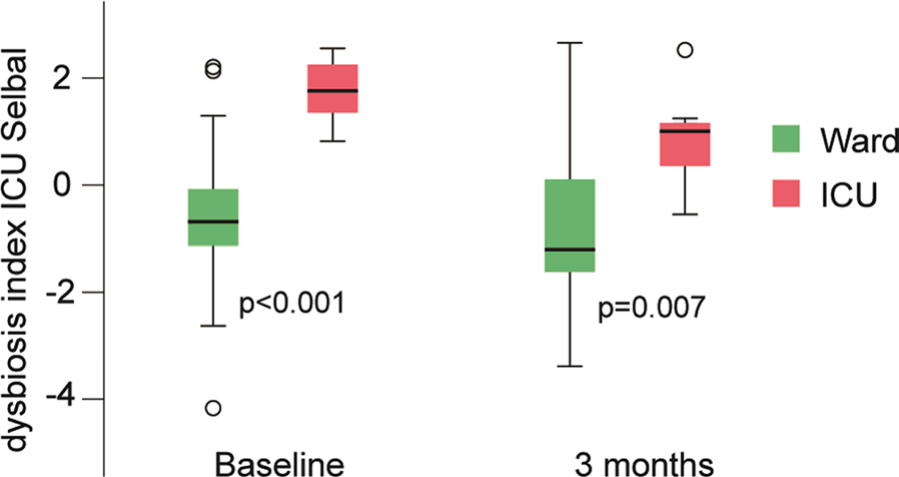
Temporal changes in dysbiosis index from baseline (within 48 h after hospital admission) to three-month post-admission (*n* = 50), depending on clinical status (ICU and ward patients) during hospitalization

As shown in Fig. 4, the ICU-related dysbiosis index remained significantly elevated in ICU patients (*n* = 7) compared with ward patients (*n* = 43) at the three-month follow-up (adjusted *p* = 0.007), although paired analysis revealed a tendency to reduction in the surviving ICU patients from baseline (*p* = 0.063). In the whole cohort, the reduction from baseline to 3 months was significant (*p* < 0.001).

#### Discussion

In this prospective Norwegian multicentre cohort of hospitalized COVID-19 patients, we assessed gut microbiota alterations in relation to need for ICU admission, and whether an ICU-related dysbiosis was associated with 60-day mortality. Our results can be summarized as follows: (i) Gut microbiota diversity was lower in COVID-19 patients requiring ICU during hospitalization than in ward patients, (ii) ICU patients had an altered gut microbiota composition (dysbiosis), independent of admission demographics that differed with ward patients, as well as antibiotic use, dexamethasone use, pulmonary comorbidities, and inflammatory markers, and (iii) the dysbiosis index was associated with 60-day mortality, also in adjusted analysis.

Several studies have reported gut microbiota alterations during acute COVID-19 and, in particular, in relation to acute respiratory failure and need of ICU [10]. Our finding of reduced microbiota diversity in ICU patients is in line with previous studies [9, 22]. Based on the ICU-related dysbiosis index, we also found that reduced relative abundance of several members of the Lachnospiraceae and Ruminococcaceae families, such as *Roseburia* and different *Eubacterium* species, which are known producers of butyrate, distinguished ICU from ward patients, also in line with several previous studies [9, 10, 22]. Butyrate has local immunomodulatory effects in the gut mucosa and is the main energy substrate for enterocytes, and is vital for gut barrier maintenance [23].

Interestingly, the ICU-related dysbiosis index also consisted of increased relative abundance of several microbes with partial affinity to the oral microbiome, including *Pyramidobacter, Olsenella* and *Rothia.* In particular, *Rothia* species have been found in increased abundance in several previous gut microbiota studies [9, 24–26] and, in one study, enriched both in the oral and gut microbiota of hospitalized COVID-19 patients [24]. Whether this dysbiosis is a consequence of viral replication in the oral cavity and/or gut mucosa [2], disrupted mucosal immunology paving the way for opportunistic pathogens, the inflammatory response or other factors [10], cannot be determined from our data. Of note, the dysbiosis index correlated with the degree of respiratory distress and markers of systemic inflammation, in line with previous studies [10]. Furthermore, data from animal models have shown that oxygen therapy could promote dysbiosis in airways and the gut that could contribute to oxygen-induced lung injury [27]. Hence, both respiratory distress and oxygen therapy itself could potentially contribute to a gut-lung axis.

Our study expands the current literature by reporting a potential link between ICU-related microbiota alterations and 60 days mortality, in line with a recent study showing an association between day-90 mortality and higher concentrations of opportunistic pathogens in the oropharyngeal and intestinal compartments of critically ill patients [13]. Of note, our data should be interpreted with caution, as our sample size was not powered for multivariate adjustment. Still, the association between dysbiosis and 60-day mortality was not weakened after separate adjustment for relevant covariates including age, comorbidities, kidney function and inflammatory markers. Treatment with dexamethasone and antibiotics attenuated the association somewhat, as these were strongly associated with 60-day mortality. For this reason, the strongest attenuation of the association between dysbiosis and 60-day mortality was seen with the ICU propensity score reflecting several parameters strongly associated with poor prognosis (e.g. dexamethasone, antibiotics and P/F-ratio).

Some of the microbes being enriched in ICU patients, including *Pyramidobacter* and *Olsenella,* also had increased relative abundance in patients who died during the first 60 days. In a previous work, enhanced gut microbiota levels of *Pyramidobacter* correlated with circulating IL-6 levels, an upstream regulator of the pro-inflammatory response in severe COVID-19 disease, and predictor of disease progression in frail adults [14] while enriched *Olsenella* were detected in the lung microbiota of COVID-19 patients [15].

60-day mortality was also associated with increased abundance of *Veillonellacea*e, *Bilophila* and *Escherichia,* known pathogens that have been reported increased in previous studies of hospitalized COVID-19 patients [10]*.* Of interest, *Veillonella* was reported enriched in both oral and faecal samples of patients with COVID-19 and was also detected in high concentration in bronchoalveolar lavage samples [28]. The oral microbiota is altered in COVID-19 patients [28, 29] and has been proposed as a potential reservoir of pulmonary co-infections, as well as contributing to a disturbed gut microbiota with potential for microbial translocation to the systemic circulation [10]. Studies of selective decontamination of the oral cavity and upper GI tract have shown promise in reducing in-hospital mortality in ICU patients receiving mechanical ventilation [30, 31]. Our data, in light of the overall literature [10], suggest that such strategies could also be relevant in future intervention trials of critical COVID-19 patients.

Although rectal swabs were collected within 48 h after hospital admission, different treatment modalities could have contributed to gut microbiota differences between ICU and ward patients. Even short courses of antibiotics may alter the gut microbiota composition, and in particular, anti-anaerobic antibiotics have been shown to increase mortality in ICU patients, possibly due to effect on gut and airway microbiota [32]. Also dexamethasone could impact the gut microbiota composition and has been reported to interfere with gut permeability [33]. Furthermore, the enrichment of a partly oral bacterial flora in the gut microbiota of ICU patients could potentially also be explained by factors such as mechanical ventilation, nasogastric feeding tubes and parenteral nutrition [34].

Whatever contributing factors, the dysbiosis index remained elevated in ICU patients compared with ward patients also at the three-month follow-up. Previous studies, including data from our own group, have shown that gut microbiota alterations can persist long-term after hospitalization and are associated with persisting pulmonary symptoms and low-grade inflammation [10, 11]. Future studies should investigate whether persistent microbiota alterations after severe COVID could affect long-term clinical outcome, including long-COVID [35].

Our study has several limitations, in particular the limited number of patients that died within 60 days, as well as the low number of ICU patients at 3-month follow-up. A formal sample size calculation was not performed, partly because there are no standardized methods for sample size calculations in microbiota studies, and the sample size was not powered for multivariate adjustment. In addition, the study lacks an independent replication cohort, and this should therefore be regarded as exploratory. Furthermore, an observational study cannot draw any conclusions regarding causation, and several factors including comorbidities, disease severity and treatment modalities could have contributed to our findings. Dietary habits before admission and nutritional support during hospitalization are potential contributors to a distorted gut microbiome, and lack of food frequency questionnaires and other data on nutrition is another limitation.

In addition, patients were included before occurrence of the current omicron strains and with a much lower vaccination coverage in the population. Hence, our findings cannot be directly extrapolated to the current epidemiological situation, as omicron strains have been shown to have lower replication in lung and gut cells compared to delta variants [36]. Our study also has obvious strengths, being one of the largest studies with microbiota sampling during acute COVID-19, repeated microbiota sampling at long-term follow-up, as well as mortality end points up to day 60.

In conclusion, our data, although exploratory, provide evidence that gut microbiota alterations detected within 48 h after hospital admission could be related to poor prognosis after severe COVID-19. To clarify the clinical impact of these findings, larger studies of gut involvement during COVID-19 in relation to long-term clinical outcome, including patients infected with current omicron variants, are warranted.

## Supplementary Information

**Additional file 1: Table S1.** Shannon diversity and ICU-related dysbiosis index according to dexamethasone and antibiotic treatment in hospitalized COVID-19 patients. **Fig. S1.** Distribution of Shannon diversity and dysbiosis index in hospitalized COVID-19 patients

## Data Availability

Regarding data sharing, Norwegian institutional data privacy regulations prohibit deposition of individual level data to public repositories. Participant written consent also does not cover public sharing of data for use for unknown purposes. However, upon contact with Marius Trøseid (marius.troseid@medisin.uio.no) or Thor Ueland (thor.ueland@medisin.uio.no), an institutional data transfer agreement can be established, and data shared if the aims of data use are covered by ethical approval and patient consent. The procedure will involve an update to the ethical approval as well as review by legal departments at both institutions, and the process will typically take 1 to 2 months from initial contact. Data will be shared via a secure online procedure.
