# Supplementary material for: Gut microbiota composition during hospitalization is associated with 60-day mortality after severe COVID-19
Source: Crit Care. 2023 Feb 23;27:69. doi: 10.1186/s13054-023-04356-2 (PMC9946863; doi:10.1186/s13054-023-04356-2)
Supplement: Supplementary file 1 — Additional file 1: Table S1. Shannon diversity and ICU-related dysbiosis index according to dexamethasone and antibiotic treatment in hospitalized COVID-19 patients. Fig. S1. Distribution of Shannon diversity and dysbiosis index in hospitalized COVID-19 patients [file 13054_2023_4356_MOESM1_ESM.docx]

**Supplemental Table 1.** Shannon diversity and ICU-related dysbiosis index according to dexamethasone and antibiotic treatment in hospitalized COVID-19 patients

|  |  | Shannon diversity | | | Dysbiosis index | | |
| --- | --- | --- | --- | --- | --- | --- | --- |
|  |  | No treatment | Treatment | *p* | No treatment | Treatment | *p* |
| Dexamethasone |  | 5.2 (4.6, 5.7) | 5.1 (4.0, 5.6) | 0.27 | -0.3 (-0.9, 0.6) | 0.7 (-0.7, 1.7) | 0.012 |
|  | Ward | 5.2 (4.6, 5.7) | 5.6 (4.9, 5.7) | 0.47 | -0.4 (-1.0, 0.3) | -0.8 (-1.2, -0.2) | 0.21 |
|  | ICU | 5.1 (4.4, 5.8) | 4.4 (3.8, 5.3) | 0.31 | 1.4 (0.9, 2.3) | 1.6 (0.9, 2.3) | 1.00 |
| Antibiotics |  | 5.2 (4.5, 5.7) | 5.1 (4.2, 5.6) | 0.46 | -0.3 (-0.9, 0.3) | 0.7 (-0.8, 1.8) | 0.002 |
|  | Ward | 5.4 (4.7, 5.7) | 5.2 (4.4, 5.7) | 0.61 | -0.4 (-0.9, 0.2) | -0.7 (-1.3, 0.2) | 0.41 |
|  | ICU | 3.5 (2.2, 4.3) | 5.0 (4.0, 5.6) | 0.058 | 0.7 (0.3, 1.4) | 1.7 (0.9, 2.4) | 0.081 |


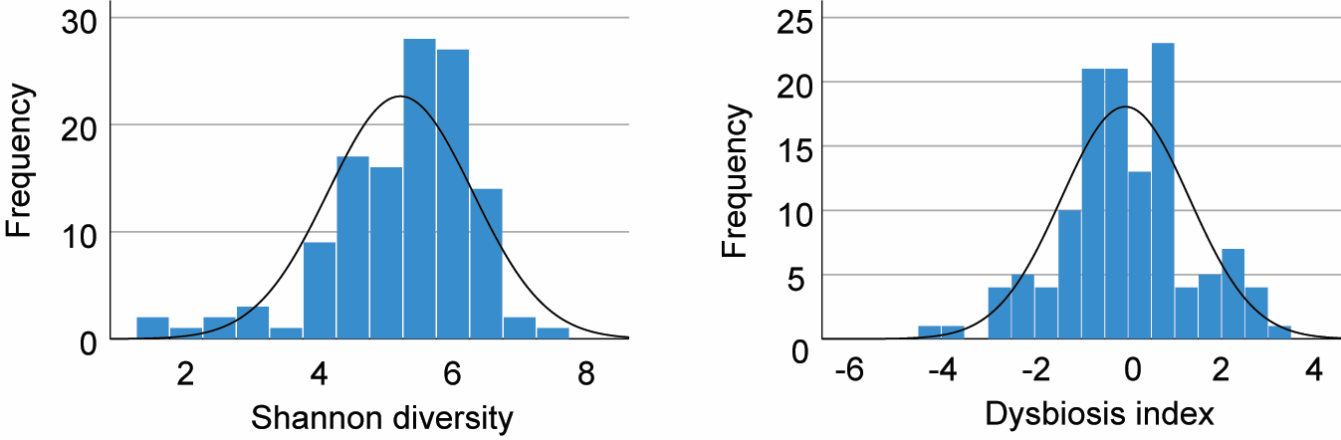


**Supplemental Figure 1.** Distribution of Shannon diversity and dysbiosis index in hospitalized COVID-19 patients
